# Fasting Increases Human Skeletal Muscle Net Phenylalanine Release and This Is Associated with Decreased mTOR Signaling

**DOI:** 10.1371/journal.pone.0102031

**Published:** 2014-07-14

**Authors:** Mikkel Holm Vendelbo, Andreas Buch Møller, Britt Christensen, Birgitte Nellemann, Berthil Frederik Forrest Clasen, K. Sreekumaran Nair, Jens Otto Lunde Jørgensen, Niels Jessen, Niels Møller

**Affiliations:** 1 Department of Endocrinology and Internal Medicine, Aarhus University Hospital, Aarhus, Denmark; 2 Research Laboratory for Biochemical Pathology, Aarhus University Hospital, Aarhus, Denmark; 3 Department of Molecular Medicine, Aarhus University Hospital, Aarhus, Denmark; 4 Division of Endocrinology, Endocrine Research Unit, Mayo Clinic, Rochester, Minnesota, United States of America; School of Medicine, University of Belgrade, Serbia

## Abstract

**Aim:**

Fasting is characterised by profound changes in energy metabolism including progressive loss of body proteins. The underlying mechanisms are however unknown and we therefore determined the effects of a 72-hour-fast on human skeletal muscle protein metabolism and activation of mammalian target of rapamycin (mTOR), a key regulator of cell growth.

**Methods:**

Eight healthy male volunteers were studied twice: in the postabsorptive state and following 72 hours of fasting. Regional muscle amino acid kinetics was measured in the forearm using amino acid tracers. Signaling to protein synthesis and breakdown were assessed in skeletal muscle biopsies obtained during non-insulin and insulin stimulated conditions on both examination days.

**Results:**

Fasting significantly increased forearm net phenylalanine release and tended to decrease phenylalanine rate of disappearance. mTOR phosphorylation was decreased by ∼50% following fasting, together with reduced downstream phosphorylation of 4EBP1, ULK1 and rpS6. In addition, the insulin stimulated increase in mTOR and rpS6 phosphorylation was significantly reduced after fasting indicating insulin resistance in this part of the signaling pathway. Autophagy initiation is in part regulated by mTOR through ULK1 and fasting increased expression of the autophagic marker LC3B-II by ∼30%. p62 is degraded during autophagy but was increased by ∼10% during fasting making interpretation of autophagic flux problematic. MAFbx and MURF1 ubiquitin ligases remained unaltered after fasting indicating no change in protesomal protein degradation.

**Conclusions:**

Our results show that during fasting increased net phenylalanine release in skeletal muscle is associated to reduced mTOR activation and concomitant decreased downstream signaling to cell growth.

## Introduction

Metabolic performance in response to fasting and caloric restriction is critical for survival and natural selection has favored physiological capacity to cope with prolonged fasting [1]. During fasting carbohydrate availability is limited and lipid represents the major energy source for substrate metabolism [2]. Amino acids derived from muscle proteins represent a storage of energy but its utilization rapidly leads to muscle wasting [2]. Instead, development of muscle insulin resistance and initiation of ketogenesis preserve glucose and minimize the need for protein breakdown during fasting [3], [4], [5], [6]. Nevertheless, fasting eventually causes a progressive loss of protein [3], [7]. Skeletal muscle contains the major human protein reservoir and consumes a major proportion of chemical energy [8], but little is known about myocellular adaptations to fasting at the molecular level in humans. Studying animal models and cell cultures have given valuable insight into the mechanisms regulating protein synthesis and breakdown. Mammalian target of rapamycin (mTOR) a central sensor of nutrient status and regulator of protein synthesis [9], [10], [11] couples energy availability to cellular homeostasis by regulating cell growth [9]. Two distinct complexes with mTOR as catalytic subunit exist: mTOR complex 1 and 2 (mTORC1 and mTORC2). Of the two complexes, mTORC1 play a crucial role in cell growth regulation [10], [12], [13]. Akt constitutes a crucial center for the anabolic action of insulin. While mTORC1 is a well-known target downstream of Akt, mTORC2 is implicated in activation of Akt through Ser^473^ phosphorylation [14], [15]. It is, however, unknown whether mTOR signaling is affected by fasting in human skeletal muscle. The present study was therefore designed to determine how fasting affects regulators of protein metabolism in human skeletal muscle. For this purpose, we combined a forearm arterio-venous amino acid kinetic technique and molecular methods assessing intramyocellular signaling in skeletal muscle in eight healthy participants after a 72-hour-fast during non-insulin and insulin stimulated conditions. We aimed to determine whether increased muscle protein loss during fasting is associated with reduced mTOR activity.

## Methods

### Ethical approval and considerations

All participants gave their written informed consent after receiving oral and written information regarding the study, according to the Declaration of Helsinki II. The ethical committee of central region Denmark approved the study.

Data from the present study on growth hormone (GH) signaling and insulin sensitivity have been published previously [16], [17].

### Study participants

Eight healthy men with no family history of diabetes participated. The average age was 26±4 years, body weight 82.9±8.8 kg and body mass index 23.8±1.6 kg/m^2^. The subjects did not take prescription medicine.

### Protocol

**Figure 1:**
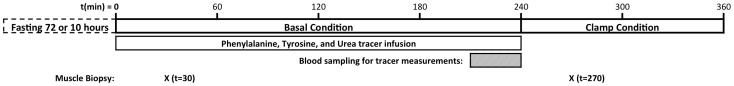
Study protocol. Subjects were examined on 2 separate days in random order: 1) after an overnight fast of 10 hours and 2) after a 72-hour fast. On both experimental days infusions of phenylalanine, tyrosine and urea tracers were initiated at t = 0, to measure amino acid kinetics during a 4 hour period (*t* = 0–240), followed by a 2-hour hyperinsulinemic euglycemic clamp (t = 240–360). At *t* = 30 and 270 min, a muscle biopsy was obtained from vastus lateralis of the quadriceps femoris muscle.

The protocol is visualized in figure 1. In a randomized crossover design, subjects were studied on 2 occasions separated by >1 month: 1) after an overnight fast of 10 hours (control), and 2) after 72 hours of fasting, during which subjects were allowed to drink tap water and to perform normal ambulatory activities, excluding physical exercise. At 0800 h (t = 0) the subjects were admitted to a quiet, thermo-neutral room on both examination days.

Intravenous catheters (Venflon; Viggo AB, Helsingborg, Sweden) were placed for infusions and blood sampling. One was placed in a dorsal hand vein for sampling, and the hand was placed in a 65°C heated box for arterialization of the blood. In the same arm, a catheter was placed in an antecubital vein for infusions. Furthermore, a third catheter was placed in the contralateral antecubital vein in a retrograde direction for deep venous samples. Before deep venous sampling the wrist cuff was inflated to a supra systolic pressure.

Subjects were studied for 6 h from 0800 to 1400 h (t = 0 to 360 min.). During the first 4 hour-basal period (t = 0–240), [^15^N]phenylalanine, [^2^H_4_]tyrosine, [^15^N]tyrosine and [^13^C]urea (Cambridge Isotope Laboratories, Andover, MA, USA) were used as amino acid tracers. The only metabolic fate of phenylalnine and tyrosine in muscle is protein synthesis and degradation [18]. After priming the amino acid pool with bolus injections of [^15^N]phenylalanine (0.7 mg/kg), [^15^N]tyrosine (0.3 mg/kg), [^2^H_4_]tyrosine (0.5 mg/kg) and [^13^C]urea (309.6 mg), continuous infusions of [^15^N]phenylalanine (0.7 mg/(kg_*_h)), [^2^H_4_]tyrosine (0.5 mg/(kg_*_h)) and [^13^C]urea (42 mg/h) were maintained for 4 hours. After 210 min. with continuous infusions, steady state was accomplished and blood samples were taken in triplicate during the last 30 min. of the period. Solutions were prepared under sterile conditions and were tested free of bacteria and pyrogens before use. Venous occlusion plethysmography was used to determine forearm blood flow.

After termination of amino acid tracer infusion glucose levels were clamped at ∼5 mM with an insulin (Actrapid, Novo Nordisk, Bagsværd, Denmark) infusion of 0.8 mU/(kg_*_min) the last 2 hours of the study (t = 240 to 360 min.). Infusions of amino acid tracers were not continued during the glucose clamp, since a 2 hour clamp may be an insufficient period of time to accomplish steady state conditions.

At t = 30 and 270 min, a 5–7 mm incision was made, using local anesthesia and sterile conditions, 12–15 cm proximal to the superior border of the patella, and a muscle specimen was obtained from the superficial border of the vastus lateralis of the quadriceps femoris muscle using the Bergström needle. The muscle tissue was immediately dissected free from fat and connective tissue and placed in liquid nitrogen.

### Blood analysis

Plasma glucose was immediately measured in duplicate on a Beckman Glucoanalyzer (Beckman Instruments, Palo Alto, CA). Serum samples were stored at −20°C. Insulin was analyzed using time resolved fluoroimmunoassay (TR-IFMA, AutoDELFIA; PerkinElmer, Turku, Finland). C-peptide was measured by ELISA (DakoCytomation, Cambridgeshire, UK). Free fatty acids (FFA) were analyzed by a commercial kit (Wako Chemicals, Neuss, Germany).

Glucagon was measured with an in-house radioimmunoassay. Free- and total-triiodothyronin (T3-free and T3-total, respectively) and cortisol were measured by ECLIA (Cobas, Roche Diagnostics Limited, West Sussex, UK). Urea was measured with UREAL assay.

### Amino acid tracers

[^15^N]phenylalanine, [^2^H_4_]tyrosine, [^15^N]tyrosine and [^13^C]urea enrichment were measured using gas chromatography-mass spectrometry (GC-MS) and concentrations of phenylalanine and tyrosine were measured (for calculation of regional amino acid kinetics) using l-[^2^H_8_]phenylalanine and l-[^13^C_6_]tyrosine as internal standards [19].

### Calculations of phenylalanine and tyrosine kinetics

Whole body phenylalanine flux (*Q*_phe_) and tyrosine flux (*Q*_tyr_) were calculated: 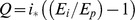
in which *i* is the rate of tracer infusion (µmol/(kg_*_h)), *E*_i_ is enrichment of the tracer infused and *E*_p_ is the plasma enrichment of the tracer at isotopic plateau [18].

The rate of phenylalanine conversion by hydroxylation to tyrosine (*I*_pt_) was calculated: 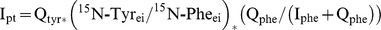
where ^15^N-Tyr_ei_ and ^15^N-Phe_ei_ are the isotopic enrichments of the respective tracers in plasma and *I*_phe_ is the infusion rate of ^15^N-phenylalanine (µmol/(kg_*_h))[20].

In this study, net phenylalanine release was calculated as follows using Fick's principle: 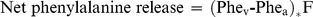
in which Phe_v_ and Phe_a_ are venous and arterial phenylalanine concentrations and *F* is blood flow in the forearm. Regional phenylalanine kinetics were calculated using the equations described by Nair et al. [19]. The arm protein breakdown represented by phenylalanine rate of appearance (Ra_phe_) was calculated: 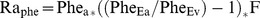
in which Phe_Ea_ and Phe_Ev_ represent phenylalanine isotopic enrichment in arterialized and venous blood, respectively [21]. The local rate of disappearance (Rd_phe_), which represents the muscle protein synthesis rate, was calculated as: 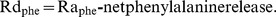



### Western blotting

Muscle biopsies were homogenized in an ice cold buffer containing (in mM) 50 HEPES, 137 NaCl, 10 Na_4_P_2_O_7_, 10 NaF, 2 EDTA, 1 MgCl_2_, 1 CaCl_2_, 2 Na_3_VO_4_, 1% (vol/vol) Nonidet P-40, 10% (vol/vol) glycerol, 2 µg/ml aprotinin, 5 µg/ml leupeptin, 0.5 µg/ml pepstatin, 10 µg/ml antipain, 1.5 mg/ml benzamidine, and 100 µM 4-(-2-aminoethyl)-benzenesulfonyl fluoride, hydrochloride (pH 7.4), and samples were rotated for 60 min at 4°C. Insoluble materials were removed by centrifugation at 16,000×g for 20 min at 4°C. Western blot analyses were used to assess protein and phosphorylation levels of various proteins. Primary antibodies against mTOR, p-mTOR(Ser^2448^), 4EBP1, non-p-4EBP1(Thr^46^), ULK1, p-ULK(Ser^757^), rpS6, p-rpS6(Ser^235-236^), TSC2, p-TSC2(Ser^1387^), eIF2α, p-eIF2α(Ser^51^), FOXO3a, p-FOXO3a(Ser^318-321^), light chain LC3B (LC3B) and β-actin were from Cell Signaling Technology (Beverly, CA, USA). p62/SQSTM1/sequestosome-1, MAFbx and MURF1 antibodies were from Abcam (Cambridge, UK).

Horseradish peroxidase (HRP)-conjugated goat antirabbit or goat antimouse IgG secondary antibody or HRP-streptavidin was used, and proteins were visualized by BioWest enhanced chemiluminescence (Pierce) and quantified using UVP BioImaging System (UVP, Upland, CA, USA). Unless otherwise mentioned, quantifications of total protein are expressed as ratio of protein to β-actin and protein phosphorylation as a ratio of total protein expression measured on the same membranes. Tissue amount only allowed measurements of 4EBP1, ULK1, MAFbx, and MURF1 in tissue from 6 out of 8 participants.

### Statistical analysis

Results are expressed as means ± SE. Normal distribution was assessed by inspection of QQ-plots, and the Levene Median test was used to test for equal variance. Isolated comparisons between control and 72 hours of fasting were assessed by a paired t-test. Comparisons between the main effects and interaction of 72 hours of fasting and insulin stimulation were assessed by a two-way repeated measurements ANOVA. When the two-way repeated-measurements ANOVA revealed significant differences, post-hoc test multiple comparison procedures using Student-Newman-Keul's method were performed. Correlations were measured using pearson product-moment correlation. P<0.05 was considered significant.

## Results

### Blood profile

During fasting FFA concentration was increased by ∼100% and glucose concentration was decreased ∼25%. These changes in metabolic substrates were accompanied with a significant decrease in the circulating levels of insulin, c-peptide and triiodothyronine, and increased glucagon levels. Comparable levels of insulin and glucose were achieved 30 minutes into the hyperinsulinemic euglycemic clamp on both occasions, but glucagon and FFA were not normalized at this time-point (Table 1).

**Table 1:** Insulin, C-peptide, glucose, FFA, glucagon, cortisol, T3-free, T3-total, and urea concentration in serum were determined at examination days during basal and clamp conditions (t = 30 and t = 270, respectively).

	Basal Condition			Clamp Condition		
	Control	Fasting	p-value	Control	Fasting	p-value
Insulin, pM	41.5±3.8	15.3±1.6	<0.001	269.0±17.5	274.5±8.7	0.74
C-peptide, pM	593±76	174±50	<0.001	426±63	276±54	<0.01
Glucose, mM	5.1±0.06	3.9±0.13	<0.001	4.9±0.19	4.6±0.07	0.29
FFA, mM	0.48±0.09	0.84±0.06	<0.01	0.27±0.03	0.72±0.09	<0.001
Glucagon, ng/L	46.4±7.2	134.5±20.1	<0.001	37.5±4.4	58.5±7.5	0.03
T3-free, pM	5.4±0.13	3.7±0.15	<0.001	5.2±0.08	3.4±0.14	<0.001
T3-total, nM	1.8±0.08	1.3±0.06	<0.001	1.7±0.05	1.1±0.05	<0.001
Cortisol, nM	456±65	503±55	0.14	369±50	414±34	0.46
Urea, mM	5.3±0.3	6.2±0.3	0.05	4.8±0.3	6.0±0.3	<0.01
Blood flow, ml/(100 ml_*_min)	2.26±42	3.45±44	0.008			
Q(phe), umol/(kg_*_h)	41.56±1.14	41.08±1.51	1			
Q(tyr), umol/(kg_*_h)	28.22±0.88	29.37±1.39	0.44			
Q(phe to tyr), umol/(kg_*_h)	3.22±0.35	2.50±0.35	0.22			
Q(urea), umol/(kg_*_h)	307±33	390±37	0.19			

Forearm blood flow, phenylalanine, tyrosine, urea and phenylalanine hydroxylation to tyrosine fluxes was measured at t = 210–240. Measurements were performed after an overnight fast of 10 hours (control) and after a 72-hour-fast (fasting). Values are means ± SE.

### Skeletal muscle amino acid kinetics

**Figure 2:**
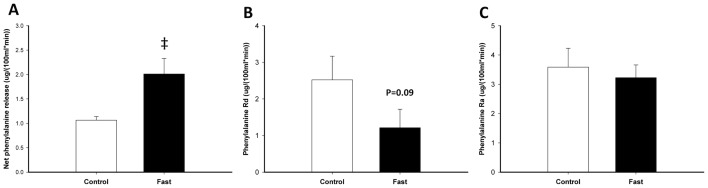
Arteriovenous phenylalanine kinetics across the forearm was assessed after an overnight fast of 10(control) and after a 72-hour-fast (fast) to determine net phenylalanine release, phenylalanine Rd, and phenylalanine Ra. Throughout the figure, open bars indicate control situation in the basal period and filled bars indicate 72: There was a significant increased forearm net phenylalanine release after 72 hours of fasting (‡ P = 0.015). B and C: The increased muscle wasting was associated with a trend (P = 0.09) toward decreased skeletal muscle protein synthesis (phenylalanine Rd) without any change in skeletal muscle protein breakdown (phenylalanine Ra).

Tracer isotopic enrichment reached steady state during the basal period of both experimental days and fasting did not impact enrichment. Blood-flow was increased in the forearm after 72 hours of fasting (Table 1) and phenylalanine kinetics across the forearm revealed a significant increase in net skeletal muscle breakdown as noted by an ∼100% increase in net phenylalanine release. The increased net skeletal muscle breakdown during fasting was associated with a trend (p = 0.09) toward decreased Rd_phe_ (skeletal muscle protein synthesis) with no change in Ra_phe_ (skeletal muscle protein breakdown) (Figure 2). Whole body phenylalanine-, tyrosine- and urea flux, and phenylalanine hydroxylation to tyrosine were unaltered after 72 hours of fasting (Table 1).

### Myocellular mTOR signaling

**Figure 3:**
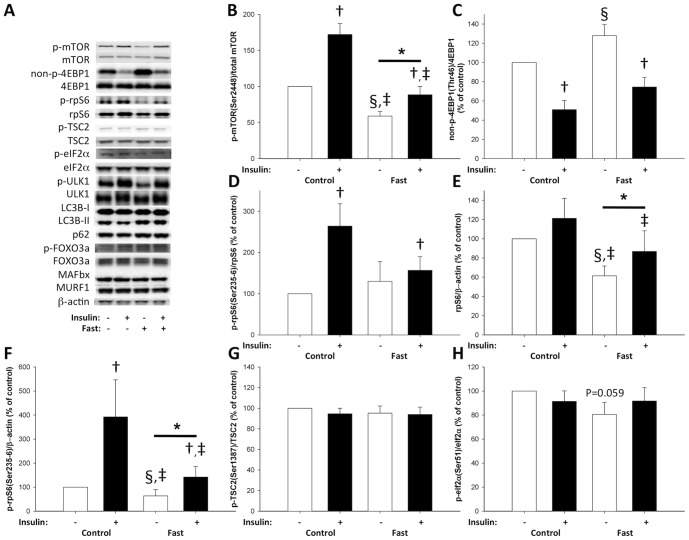
mTOR signaling pathway. A: Representative blots show from *left* to *right* control situation before and after insulin stimulation and fasting conditions before and after insulin stimulation. Phosphorylated (p), non-phosphorylated (non-p), and total protein expression. Total protein expression and phosphorylation of mTOR, 4EBP1, rpS6, TSC2, and eIF2α, from muscle biopsies taken after an overnight fast of 10 hours (control) and after a 72-hour-fast (fast) before and during a hyperinsulinemic euglycemic clamp, were assessed with western blotting. Throughout the figure, open bars indicate no insulin stimulation and filled bars insulin stimulation. B: Phosphorylation of mTOR was significantly decreased after 72 hours fasting (§ P<0.05). Furthermore, there was a main effect of 72 hours of fasting (* P<0.05) and insulin stimulation on mTOR Ser^2448^ phosphorylation († P<0.05). Post hoc test showed that mTOR phosphorylation before and during insulin stimulation was significantly decreased (‡ P<0.05). C: Phosphorylation of 4EBP1 Thr^46^ was significantly decreased after 72 hours fasting, seen by increased non-phosphorylated 4EBP1 Thr^46^ (§ P<0.05). Furthermore, there was a main effect of insulin stimulation on 4EBP1 Thr^46^ phosphorylation († P<0.05). D: There was a main effect of insulin stimulation on rpS6 Ser^235-236^ phosphorylation († P<0.05) when normalized to total rpS6 protein expression. E: Total rpS6 protein expression was significantly decreased after 72 hours fasting (§ P<0.05). There was a main effect of 72 hours fasting (* P<0.05) on rpS6 protein expression and post hoc test showed that control vs. fasting before and during insulin stimulation was significantly decreased (‡ P<0.05). F: There was a main effect of 72 hours fasting (* P<0.05) and insulin stimulation on rpS6 Ser^235-236^ phosphorylation († P<0.05) when normalized to β-actin. Post hoc test showed that rpS6 phosphorylation before and during insulin stimulation was significantly decreased (‡ P<0.05). G: 72 hours of fasting and insulin stimulation did not affect TSC2 phosphorylation at the AMPK target site Ser^1387^. H: Fasting and insulin stimulation revealed a significant interaction of eIF2α Ser^51^ phosphorylation (p = 0.048). Post-hoc test showed a trend (p = 0.059) between phosphorylation levels after 72 hours fasting in basal condition.

**Figure 4:**
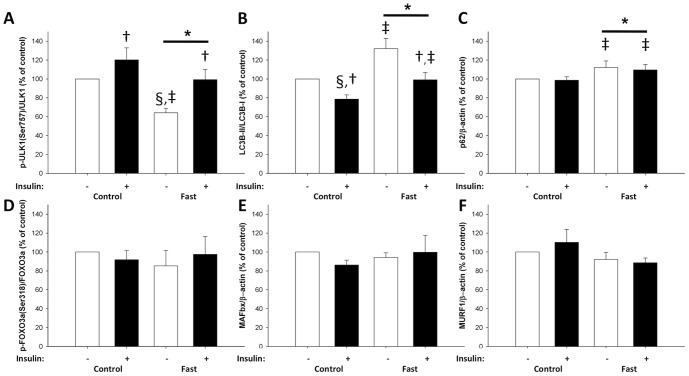
ULK1, LC3B, p62, FOXO3a, MAFbx, and MURF1 levels in skeletal muscle biopsies taken after an overnight fast of 10(control) and after a 72-hour-fast (fast) before and during a hyperinsulinemic euglycemic clamp were assessed with western blotting. Throughout the figure, open bars indicate no insulin stimulation and filled bars insulin stimulation. A: Phosphorylation of ULK1 was significantly decreased after 72 hours fasting (§ P<0.05). Furthermore, there was a main effect of 72 hours of fasting (* P<0.05) and insulin stimulation on ULK1 Ser^757^ phosphorylation († P<0.05). Post hoc test showed that ULK1 phosphorylation in basal condition was significantly decreased after fasting (‡ P<0.05). B: there was a main effect of 72 hours of fasting (* P<0.05) and insulin stimulation († P<0.05) on LC3B-II expression. Post hoc test showed that LC3B-II before and during insulin stimulation was significantly increased (‡ P<0.05). C: there was a main effect of 72 hours of fasting on p62 protein expression (* P<0.05). Post hoc test showed that p62 expression was significantly increased both during the basal period and during the hyperinsulinemic euglycemic clamp (‡ P<0.05), without any effect of insulin stimulation. D, E, and F: 72 hours of fasting and insulin stimulation did not affect FOXO3a Ser^318-321^ phosphorylation or protein expression of MAFbx and MURF1.

Fasting significantly reduced phosphorylation of the activating site Ser^2448^ on mTOR by ∼40% (p<0.05). Insulin stimulated mTOR phosphorylation on both examination days, but the increase during fasting was substantially reduced (Figure 3B). To test whether the reduced mTOR phosphorylation was associated with reduced mTOR complex 1 (mTORC1) signaling we examined downstream signaling to eukaryotic translation initiation factor 4E binding protein 1 (4EBP1), UNK-51-like kinase 1 (ULK1) and ribosomal protein S6 (rpS6). After 72 hours of fasting phosphorylation was significantly decreased on both 4EBP1 Thr^46^ (observed by increased non-phosphorylated 4EBP1) and ULK1Ser^757^ (Figure 3C and 4A). The ratio of phosphorylated vs. total rpS6 expression was unaltered after fasting (Figure 3D). However, total rpS6 protein expression was significantly reduced, and in accordance with the reduced mTOR phosphorylation, rpS6 Ser^235-236^ phosphorylation expressed as ratio to β-actin was decreased (Figures 3E and 3F).

Insulin increased phosphorylation on all examined target-sites downstream of mTORC1 (Figure 3B, 3C, 3D, 3F, and 4A). Furthermore, changes in mTOR Ser^2448^ phosphorylation correlated with changes in downstream phosphorylation (non-phospho-4EBP1(Thr^46^) Correlation Coefficient (CC) = −0.67, p = 0.005; ULK1(Ser^757^) CC = 0.58, p = 0.017; rpS6(Ser^235-236^)/β-actin CC = 0.51, p = 0.012). In addition, mTOR phosphorylation was correlated (CC = 0.41, p = 0.048) with Ser^473^ phosphorylation on previously examined Akt [16].

We next examined modulators of mTOR pathway signaling. Tuberous sclerosis 2 (TSC2) phosphorylation at the AMP-activated protein kinase (AMPK) target site Ser^1387^ was not altered by fasting or insulin stimulation (Figure 3G). Eukaryotic factor 2 subunit α (eIF2α Ser^51^ phosphorylation showed significant interaction (p = 0.048) between fasting and insulin stimulation after the 72-hour-fast and post-hoc testing showed a trend (p = 0.059) toward decreased phosphorylation after 72 hours of fasting (Figure 3H). In addition, no changes were observed in venous phenylalanine concentrations (Control: 7.75±0.91 vs. Fast: 7.89±0.75 mg/L, p = 0.59).

ULK1 is essential for autophagy and is inhibited by mTORC1 through phosphorylation on Ser^757^. Consequently, we examined markers of autophagy and fasting increased light chain 3 (LC3)B-II protein content by ∼30% compared to LC3B-I. Insulin stimulation reduced LC3B-II levels on both experimental days with no effects of fasting (Figure 4B). We also determined p62 protein expression which was slightly (∼10%) but significantly increased by fasting and unaltered by insulin stimulation (Figure 4C). Forkhead box O3a (FOXO3a) Ser^318-321^ phosphorylation was not affected by fasting or insulin stimulation (Figure 4D). MAFbx and MURF1 marks proteins for proteasomal degradation in skeletal muscle, but the protein expression of both proteins were unaltered after both fasting and insulin stimulation (Figure 4E and 4F).

## Discussion

The current study demonstrates that increased skeletal muscle net phenylalanine release following a 72-hour-fast is associated with decreased mTOR activity. The mTORC1 complex is a key regulator of cell growth and anabolism, and has been extensively studied in cell lines and during different conditions of nutrient availability [10]. Hypertrophy and atrophy of rat plantaris muscle is tightly coupled to alterations in mTORC1 activity [22]. In humans, skeletal muscle hypertrophy is accompanied by a significant increase in mTORC1 activity whereas reduced protein synthesis is characterized by decreased mTORC1 activity [23], [24], [25]. After 72 hours of fasting we found increased net phenylalanine release (net skeletal muscle breakdown) partly driven by increased limb blood flow as described previously [6]. We furthermore found a trend (p = 0.09) towards reduced skeletal muscle protein synthesis (Rd_phe_) in parallel with decreased mTOR Ser^2448^ phosphorylation. This phosphorylation is essential for mTORC1 downstream regulation of ribosomal mRNA translation [9], [11]. We therefore examined 4EBP1 and ULK1 phosphorylation sites directly downstream of mTORC1 [9], [26], and both were significantly reduced after 72 hours fasting and correlated with the mTOR phosphorylation pattern. The highly energy consuming process of ribosomal biogenesis is tightly coupled to energy availability through mTORC1 mediated up-regulation of nuclear RNA polymerases transcription activities in yeast and mammal-cells [27], [28], and this could explain the observed reduction in rpS6 protein expression after 72 hours fasting. Furthermore, mTORC1 regulates rpS6 Ser^235-236^ phosphorylation through ribosomal protein S6 kinase of 70 kDa (p70S6K), and this phosphorylation site was also decreased after 72 hours fasting when expressed as ratio of β-actin. The reduced phosphorylation on mTOR Ser^2448^ together with decreased phosphorylation on all examined downstream mTORC1 target sites clearly indicate reduced activity of this complex after fasting. The decreased mTORC1 activity is in accordance with the observed increase in amino acid release from the forearm and the trend towards reduced phenylalanine rate of disappearance, as well as previous studies describing mTORC1 and protein metabolism in other models [9].

Even though no fasting effect was observed on the validated mTORC2 downstream target site, Akt Ser^473^, a positive correlation between mTOR Ser^2448^ and Akt Ser^473^ phosphorylation was observed. This positive correlation indicates a connection between the regulation of these two proteins during fasting and insulin stimulation. Our study does not define the hormonal mechanisms regulating the observed alterations of intramyocellular protein metabolic signaling. However, insulin inhibits muscle protein breakdown and activates Akt and mTOR phosphorylation [29], so hypoinsulinaemia during fasting may potentially contribute to the increased net phenylalanine release. During insulin stimulation Akt Ser^473^ and mTOR Ser^2448^ phosphorylation was increased on both examination days. Interpretation of the hierarchical order of these phosphorylations is impossible because skeletal muscle biopsies only were obtained once during insulin stimulation.

Insulin stimulated phosphorylation on mTOR Ser^2448^ and rpS6 Ser^235-236^ (expressed as ratio of β-actin) did not respond to the same extend after 72 hours fasting as after the overnight fast, indicating insulin resistance in this part of the insulin signaling pathway. Consequently, we examined modulators of the signal downstream of Akt. To elucidate whether the reduced phosphorylations could be explained by changes in AMP/ATP ratio, we also examined the AMPK target site, upstream of mTORC1, Ser^1387^ on TSC2 [30], [31], and observed no changes of TSC2 Ser^1387^ phosphorylation. This is in accordance with the previously reported unaltered AMPK activity after 72 hours fasting [16]. eIF2α reduce global mRNA translation when phosphorylated upon diverse stress signals, including amino acid deprivation[32]. Given that amino acid deprivation also inhibits protein synthesis and mTORC1 activity in cell lines [33], we further examined eIF2α Ser^51^ phosphorylation. If anything – we found a trend towards decreased phosphorylation of eIF2α after 72 hours fasting, indicating that the increased net phenylalanine release is not due to eIF2α repression of translation. It can be assumed that venous phenylalanine concentrations reaches equilibrium with intramyocellular concentrations across the capillary bed. The present unchanged venous phenylalanine concentrations and absence of increased eIF2α phosphorylation after 72 hours fasting suggests that the decreased mTORC1 activity is not due to decreased amino acid concentrations.

Autophagy is a catabolic process that delivers proteins to the lysosomes for degradation and is regulated in response to changing nutrient and energetic conditions. Studies in mice indicate a pivotal role for autophagy in surviving short periods without nutrient supply [34]. At the molecular level, autophagy initiation is enzymatically regulated by mTORC1 [35], [36]. Inhibition of mTORC1 leads to transcription-independent up-regulation of autophagy, seen by LC3B-I conversion to LC3B-II through lipidation [37]. In accordance with the fasting-induced reduction of mTORC1 activity, we found that LC3B-II content was significantly increased, suggesting increased autophagosome appearance. In addition to this, we observed a fasting-induced reduction in ULK1 Ser^757^ phosphorylation. Phosphorylation of ULK1 at Ser^757^ has previously been reported as a direct mTORC1 phoshorylation site involved in mTORC1 mediated suppression of autophagy [26]. These data suggests that mTORC1 mediated suppression of ULK1 Ser^757^ phosphorylation as a mechanism by which autophagy is activated in response to fasting. AMPK has also been shown to increase autophagy by activating ULK1 through phosphorylation of distinct sites [26]. However, as previously reported [16], we did not observe any changes in AMPK phosphorylation in the present study, suggesting no involvement of this mechanism in the fasting response of human skeletal muscle. Given that LC3B-II levels not per se measure autophagic flux [37], [38], we also determined p62 protein expression. p62 is degraded in the autophagic process and inhibition of autophagy leads to p62 accumulation [37], [39]. We observed slightly (10%), but significantly, increased p62 levels in response to fasting. This contradicts our LC3B findings by suggesting inhibited autophagy. The quantitative data suggest that the changes in p62 protein expression are not as pronounced as for LC3B-II. Never the less, we are not able to exclude to possibility that the elevated LC3B-II levels in the fasted state reflects autophagosome accumulation and autophagy inhibition rather than autophagy induction. Insulin stimulation reduced LC3B-II levels equally on both experimental days, suggesting that LC3B-II formation and autophagy induction is impeded during insulin stimulation, while LC3B-II degradation continues at equal rate as in basal conditions. In line with this finding, we observed increased phosphorylation of ULK1 at Ser^757^ during insulin stimulation, suggesting mTORC1 mediated phosphorylation of ULK1 as a potential mechanism by which insulin suppresses autophagy in human skeletal muscle. However, p62 levels remained unaltered during insulin stimulation, and we are therefore unable to determine whether or not autophagic flux is affected. In addition to enzymatic control, autophagy has also been shown to be transcriptionally regulated by forkhead box O (FOXO) 3a through a mechanism phosphorylating Ser^318^ [40]. In the present study, FOXO3a Ser^318^ phosphorylation remained unaltered in response to fasting and insulin stimulation. This finding either suggests that the divergence between our LC3B-II and p62 findings reflects unaltered autophagic flux, or that the autophagic response to fasting and insulin are independent of FOXO3a Ser^318^ phosphorylation. Collectively, our data suggest that LC3B lipidation is controlled by a mechanism that involves mTORC1 mediated phosphorylation and dephosphorylation of ULK1 at Ser^757^ in response to fasting and insulin stimulation in human skeletal muscle. However, due to the conflicting nature of the data used to determine autophagic flux, we are unable to associate these findings to the increased skeletal muscle net phenylalanine release following a 72-hour-fast.

In parallel with autophagy being essential for mice to survive nutrient deprivation, fasted mice do also show upregulation of ubiquitin protein E3-ligase increased proteasome mediated protein degradation [41], and muscle loss is attenuated if MAFbx activity is inhibited [42]. Specially, MAFbx increases in mice with fasting [43]. In humans increased gene expression of the two E3 ubiquitin ligases, MAFbx and/or MURF1, is associated to atrophy following immobilization [44], [45], [46], [47] and early after spinal cord injury [48]. However, increased mRNA is not always reported after unloading [49] and mRNA levels do not necessarily reflect protein expression in these studies [48]. Even though no change in Ra_phe_ (skeletal muscle protein breakdown) was observed after 72 hours fasting we measured MAFbx and MURF1 protein expression, because amino acids from increased protein breakdown could be used for incorporation in newly synthesized proteins and therefore escape detection by the tracer dilution method employed. However, in accordance with previously reported unchanged MAFbx and MURF1 mRNA levels in human skeletal muscle after fasting for 40 hours [50], we did not observe any effect of 72 hours fasting on protein expression of MAFbx or MURF1 in present study.

Arteriovenous amino acid kinetics across muscle beds assessing net muscle breakdown, rate of protein synthesis and protein breakdown have been used to define the muscle response to fasting but have produced conflicting results. It has been reported that net muscle amino acid release increases with fasting [4], due to increased skeletal muscle protein breakdown without affecting skeletal muscle protein synthesis [5]. In the present study net phenylalanine release was increased maybe due to decreased protein synthesis. In contrast, others have failed to demonstrate these changes in regional protein metabolism [6], [51]. In present study the combination and concordance of tracer kinetics and measurements of myocellular signaling therefore further strengthens our understanding of fasting induced skeletal muscle wasting.

In conclusion, by use of a phenylalanine tracer we observed increased net skeletal muscle breakdown and a trend towards a decrease in human skeletal muscle protein synthesis after 72 hours fasting. This was associated with reduced mTOR activation and concomitant decreased downstream signaling to cell growth.
